# Ovine *KRTAP36-2*: A New Keratin-Associated Protein Gene Related to Variation in Wool Yield

**DOI:** 10.3390/genes14112045

**Published:** 2023-11-06

**Authors:** Huitong Zhou, Wenhao Li, Lingrong Bai, Jiqing Wang, Yuzhu Luo, Shaobin Li, Jonathan G. H. Hickford

**Affiliations:** 1 International Wool Research Institute, Gansu Agricultural University, Lanzhou 730070, China; huitong.zhou@lincoln.ac.nz (H.Z.); wangjq@gsau.edu.cn (J.W.); luoyz@gsau.edu.cn (Y.L.); 2 Gene-Marker Laboratory, Faculty of Agricultural and Life Sciences, Lincoln University, Lincoln 7647, New Zealand; lingrong.bai@lincolnuni.ac.nz; 3 Plateau Livestock Genetic Resources Protection and Innovative Utilization Key Laboratory of Qinghai Province, Key Laboratory of Animal Genetics and Breeding on Tibetan Plateau, Ministry of Agriculture and Rural Affairs, Qinghai Academy of Animal Science and Veterinary Medicine, Qinghai University, Xining 810016, China; qhdxlwh@163.com; 4 Gansu Key Laboratory of Herbivorous Animal Biotechnology, College of Animal Science and Technology, Gansu Agricultural University, Lanzhou 730070, China

**Keywords:** keratin-associated protein, KAP36-2, sheep, wool yield

## Abstract

Keratin-associated proteins (KAPs) are structural components of wool fibres. High-glycine/tyrosine (HGT)-KAPs are a subset of the KAP family, and their abundance in fibres varies. In this study, we report the discovery of an ovine HGT-KAP gene to which we assigned the name *KRTAP36-2*. Polymerase chain reaction and single-strand conformation polymorphism (PCR-SSCP) analyses revealed four variants of this gene in a screening population of 170 sheep from a variety of breeds. The DNA sequencing of the variants revealed four single-nucleotide polymorphisms (SNPs) and a dinucleotide deletion. Three of these SNPs were in the coding region, and one of these was non-synonymous and potentially led to the amino acid substitution p.Cys27Gly near the middle of the protein. The remaining SNP was located near the putative TATA box, and the di-nucleotide deletion was near the putative transcription initiation site. The effect of this variation in *KRTAP36-2* was investigated in 274 Southdown × Merino lambs that were the progeny of five sires. Variation was only found to be associated with wool yield, that is, the proportion of the greasy fleece that remained as clean fleece upon scouring (expressed as a percentage). This may have some value in increasing wool production.

## 1. Introduction

Wool is a natural fibre produced by follicles in the skin of sheep. The fibre is primarily composed of keratin (K) proteins and keratin-associated proteins (KAPs), with the former being built into the keratin intermediate filaments and the latter forming an inter-filamentous protein matrix that embeds the keratin intermediate filaments [1] and is covalently bound to the keratins.

Keratins comprise type I (acidic) and type II (basic/neutral) proteins, and KAPs have historically been classified as consisting of high-sulphur (HS), ultra-high-sulphur (UHS), and high-glycine/tyrosine (HGT) KAPs [1]. The number of keratins in wool seems to be comparable to that described for humans, with ten type I and seven type II sheep keratins [2] compared to eleven type I and six type II hair keratins in human hair [3].

The KAPs are more diverse, with 88 members comprising 24 HS-, 47 UHS-, and 17 HGT-KAPs described in humans [4,5,6]. A full list of the KAPs in sheep is far from complete, but they appear to have more than humans, as four additional KAP genes (designated as *KRTAPs*) have been reported in the sheep genome, namely *KRTAP6-4*, *KRTAP6-5*, *KRTAP8-2*, and *KRTAP36-1* [7,8]. All these additional genes encode HGT-KAPs, and this suggests a difference in the HGT-KAP profile between wool and human hair.

The HGT-KAPs are described as the first group of KAPs expressed after keratin intermediate filament production, and they are mainly present in the orthocortex of wool fibres [9]. The content of HGT-KAPs in fibres varies between and within species, ranging from less than 3% in human hair and Lincoln sheep wool through to 4–12% in Merino sheep wool, 18% in the hair of mice, and 30–40% in echidna quills [10]. The expression of the HGT-*KRTAPs* was found to be down-regulated in the wool follicles of felting lustre mutant sheep [11], and variation in the HGT-*KRTAPs* was reported to be associated with different wool traits in sheep [7,12,13] and goats [14,15]. These findings support the contention that HGT-*KRTAPs* play a part in influencing wool fibre properties.

Fourteen HGT-*KRTAPs* have been identified in sheep to date, and all of these were in a region of approximately 721 kb between two HS-*KRTAPs* (*KRTAP11-1* and *KRTAP15-1*) on chromosome 1 [16]. In this study, we report the discovery of a new HGT-*KRTAP* that is closely related to the recently identified HGT-*KRTAP36-1* [8]. Nucleotide sequence variation in this new *KRTAP* was revealed, and the effect of this sequence variation on wool characteristics was assessed in Southdown × Merino sheep.

## 2. Materials and Methods

### 2.1. The Sheep Examined and the Collection of Wool Samples

Ethical approval was waived for this study, because the collection of sheep blood drops by the nicking of their ears is covered by Section 7.5 Animal Identification in *Code of Welfare: Sheep and Beef Cattle* (2016) issued under the Animal Welfare Act 1999 (New Zealand Government). Wool sampling at shearing is a standard industry practice.

Two groups of sheep were examined. The first group comprised 170 sheep from a variety of sheep breeds in New Zealand, including Awassi (n = 10), Coopworth (n = 10), Corriedale (n = 10), Dorper (n = 10), Perendale (n = 10), Poll Dorset (n = 10), Merino (n = 30), Romney (n = 30), Southdown (n = 30), Suffolk (n = 10), and Texel (n = 10). These sheep were randomly selected from 34 different farms and were used for variation screening.

The second group comprised 274 Southdown × Merino lambs descended from five sires. These were used for the association analyses. The lambs were tagged with a unique identification number at birth that identified them by their mother and the sire she was mated to, and their gender, birth date, birth weight, and birth rank (i.e., whether they were an individual, twin, or triplet) were recorded. These lambs were shorn at twelve months of age. At shearing, the greasy fleece weight (GFW) was quantified for each lamb, and a sample of their wool was collected from the mid-side region for wool trait measurement using International Wool Textile Organisation (IWTO) standardised methods.

Testing was undertaken at the New Zealand Wool Testing Authority Ltd. (NZWTA, Napier, New Zealand) using recognised IWTO standardised procedures. First, the IWTO scoured wool yield (YIELD) at 16% regain (moisture content) was calculated by determining the weight of the wool sample before and after scouring. YIELD is essentially the proportion of the greasy fleece that remained as clean fibre expressed as a percentage. Other IWTO-recognised phenotypic measurements included the mean staple length (MSL), mean fibre diameter (MFD), mean fibre curvature (MFC), fibre diameter standard deviation (FDSD), mean staple strength (MSS), coefficient of variation of fibre diameter (CVFD), and prickle factor (PF-the percentage of fibres with a diameter greater than 30 microns). From the yield measurements, the clean fleece weight (CFW) for the whole fleece could then be calculated.

A sample of blood from each sheep was collected on TFN paper (Munktell Filter AB, Falun, Sweden), and genomic DNA was purified with the two-step washing procedure detailed in Zhou et al. [17].

### 2.2. PCR Amplification

An ovine *KRTAP36-1* sequence MK770622 was used to blast the sheep genome assembly ARS-UI_Ramb_v2.0 (NC_056054.1) to identify any homologous sequence containing an open reading frame (ORF) that would encode a putative KAP protein. Once identified, sequences flanking the ORF were used to design PCR primers (5′-TGGTTTACCACACCCACATG-3′ and 5′-TCATTTTGAAGCAAGCGATAG-3′) that would amplify the whole presumed coding sequence, and these were synthesised by Integrated DNA Technologies (Coralville, IA, USA). The predicted amplicon size was 363 base pairs.

The PCR amplification of this ORF was performed in a 15 μL reaction containing the genomic DNA on one 1.2 mm punch of TFN paper; 0.25 μM of each primer; 150 μM of each dNTP (Eppendorf, Hamburg, Germany); 2.5 mM of Mg^2+^; 0.5 U of Taq DNA polymerase (Qiagen, Hilden, Germany); and 1× the reaction buffer supplied with the enzyme. The thermal profile consisted of an initial denaturation for 2 min at 94 °C; followed by 35 cycles of 30 s at 94 °C, 30 s at 60 °C, and 30 s at 72 °C; and a final extension of 5 min at 72 °C. The PCR amplifications were undertaken in S1000 thermal cyclers (Bio-Rad, Hercules, CA, USA).

### 2.3. Screening for Sequence Variation in the Amplicon and DNA Sequencing

The PCR amplicons obtained were subject to single-strand conformation polymorphism (SSCP) analysis to screen for sequence variation. A 0.7 µL aliquot of each amplicon was mixed with 7 µL of loading dye (98% formamide, 10 mM EDTA, 0.025% bromophenol blue, 0.025% xylene-cyanol). After amplicon denaturation at 95 °C for 5 min, the amplicons in the loading dye were cooled in wet ice and then loaded immediately on 16 cm × 18 cm 14% acrylamide:bisacrylamide (37.5:1) (Bio-Rad) gels. Electrophoresis was performed using Protean II xi cells (Bio-Rad) at 310 V for 20 h and 18 °C in 0.5× TBE buffer. The gels were silver-stained using the method described by Byun et al. [18].

Amplicons representative of different SSCP patterns from sheep that appeared to be homozygous in the amplified region were sequenced using a Sanger dideoxy method and the same PCR primers at the Lincoln University DNA Sequencing Facility. These are typically simple patterns with just two bands. Rarer variants that were only found in heterozygous sheep (typically producing four SSCP gel bands) were sequenced using a rapid approach that has been described previously [19]. In this approach, a band corresponding to the rare variant was excised as a gel slice from the polyacrylamide gel, macerated, and then used as a template for re-amplification with the original primers. This second amplicon was then sequenced at the Lincoln University DNA Sequencing Facility.

### 2.4. DNA Sequence Analyses

The compilation and alignment of the DNA sequences, their translation into putative amino acid sequences, and the construction of a phylogenetic tree were carried out using DNAMAN (version 5.2.10, Lynnon BioSoft, Vaudreuil, QC, Canada). The BLAST algorithm was used to search the NCBI GenBank (www.ncbi.nlm.nih.gov/, accessed on 14 April 2023) databases for homologous sequences.

### 2.5. Statistical Analyses of Associations

Statistical analyses were undertaken with Minitab version 16 (Minitab Inc., State College, PA, USA). General linear mixed-effect models (GLMMs) were used to evaluate the effect of the absence or presence of individual variants on the wool characteristics that had been measured or calculated. In the preliminary ANOVAs, gender and sire were found to affect (*p* < 0.05) all the wool traits, and so they were included in the models as fixed and random factors, respectively. Birth rank was not found to affect wool traits, and thus it was not entered into the models.

The model was: Y_jkl_ = µ + V_j_ + G_k_ + S_l_ + e_jkl_, where Y_jkl_ is the trait marginal mean, µ is the group raw mean for the trait, V_j_ is the effect of the jth variant (presence and absence), G_k_ is the effect of gender, S_l_ is the effect of the lth sire, and e_jkl_ is the random residual effect. Unless denoted, all *p* values were deemed significant when *p* < 0.05, and trends were logged when 0.05 ≤ *p* < 0.10.

## 3. Results

### 3.1. A Newly Identified Ovine HGT-KRTAP

**Figure 1:**
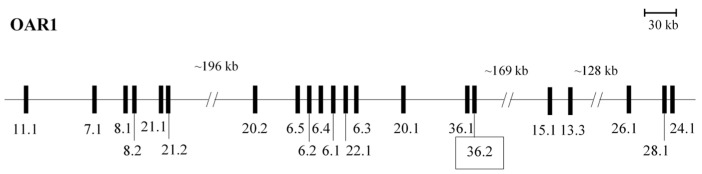
The clustering of *KRTAP36-2* within the 20 previously identified *KRTAPs* on ovine chromosome 1. The newly identified gene *KRTAP36-2* is shown in the box close to *KRTAP36-1*. The locations of the *KRTAPs* are presented as vertical bars, with their names given in an abbreviated form under the bars (i.e., *KRTAP11-1* is shown as 11.1). The nucleotide distances are only approximate, and they were derived from the sequence NC_056054.1.

A BLASTN search of the sheep ARS-UI_Ramb_v2.0 genome assembly using the ovine *KRTAP36-1* sequence MK770622 as the search sequence revealed two homologous ORFs on chromosome 1: NC_056054.1 nt125899040_125899213 (identity 100%, e-value 3 × 10^−86^) and nt125907093_125907266 (identity 89%, e-value 1 × 10^−51^). The first ORF was, not unexpectedly, ovine *KRTAP36-1* itself, while the second was located approximately 7.9 kb downstream of *KRTAP36-1*. This meant it would be clustered in a region with another 20 previously identified *KRTAPs* (Figure 1).

**Figure 2:**
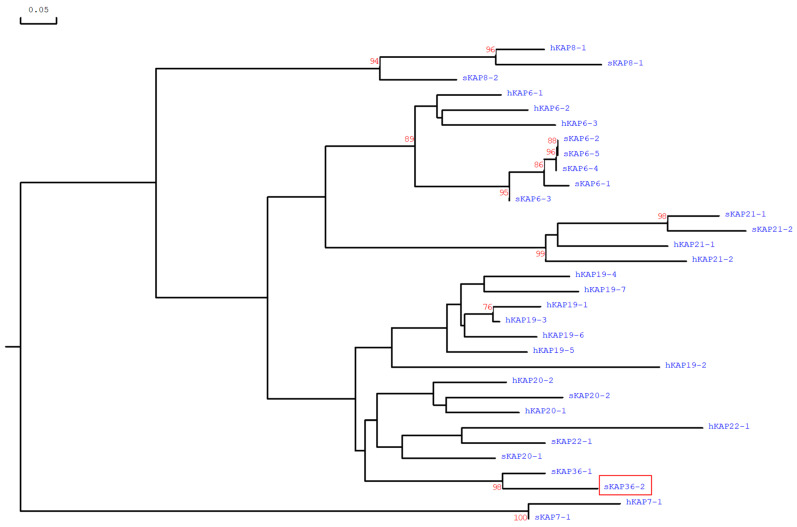
A phylogenetic tree created based on the anticipated amino acid sequences for ovine KAP36-2 and all the other high-glycine-tyrosine *KRTAPs* detected in sheep and humans to date. The numbers at the forks denote the bootstrap confidence values and only those equal to or higher than 70% are presented. The scale bar indicates a rate of 0.05 amino acid substitutions per site. The ovine and human sequences are identified with the prefixes “s” and “h”, respectively. The newly identified ovine KAP36-2 sequence is shown in a red frame. Other sequences were retrieved from GenBank with accession numbers: NM_001193399 (sKAP6-1), KT725832 (sKAP6-2), KT725837 (sKAP6-3), KT725840 (sKAP6-4), KT725845 (sKAP6-5), X05638 (sKAP7-1), X05639 (sKAP8-1), KF220646 (sKAP8-2), MH243552 (sKAP20-1), MH071391 (sKAP20-2), KX377616 (sKAP22-1), MK770620 (sKAP36-1), NM_181602 (hKAP6-1), NM_181604 (hKAP6-2), NM_181605 (hKAP6-3), AJ457063 (hKAP7-1), AJ457064 (hKAP8-1), AJ457067 (hKAP19-1), NM_181608 (hKAP19-2), NM_181609 (hKAP19-3), NM_181610 (hKAP19-4), NM_181611 (hKAP19-5), NM_181612 (hKAP19-6), NM_181614 (hKAP19-7), NM_181615 (hKAP20-1), NM_181616 (hKAP20-2), NM_181619 (hKAP21-1), NM_181617 (hKAP21-2), and NM_181620 (hKAP22-1).

This new ORF would encode a glycine-tyrosine-rich (57.9 mol%) protein if expressed and translated. The predicted amino acid sequence of this ORF was phylogenetically different to those of all other reported HGT-*KRTAPs* identified in humans and sheep, and the closest similarity was with *KRTAP36-1* (Figure 2). This ORF was therefore named *KRTAP36-2*, reflecting both its chromosomal location and its nucleotide sequence. The amino acid sequence would differ from the assumed KAP36-1 sequence, in that the KAP36-2 protein would contain 3.5 mol% cysteine, while KAP36-1 lacks cysteine.

A subsequent BLASTN search of the NCBI Expressed Sequence Tag (EST) database revealed that *KRTAP36-2* had a sequence identity of 99% to 19 ovine ESTs derived from skin tissue/wool follicles (see Supplementary Table S1), suggesting that *KRTAP36-2* is expressed in the wool follicle and that the minor sequence differences observed between the gene and mRNAs reflects minor nucleotide sequence differences in the gene.

### 3.2. Nucleotide Sequence Variation in Ovine KRTAP36-2

**Figure 3:**
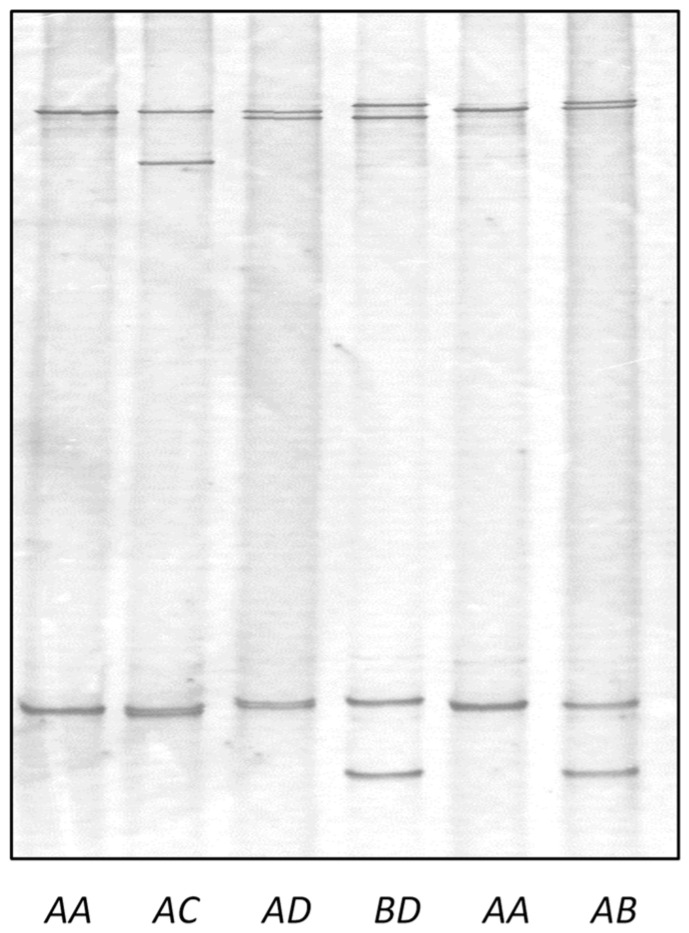
PCR-single strand conformation polymorphism of ovine *KRTAP36-2*. Four unique banding patterns representing four different variant sequences (*A*, *B*, *C*, and *D*) are shown in either homozygous or heterozygous forms.

**Figure 4:**
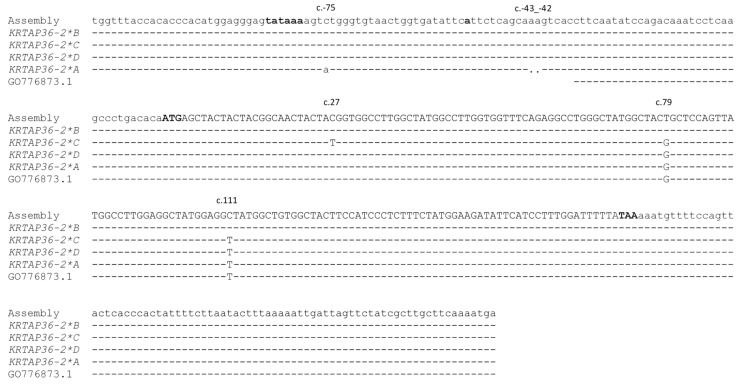
Sequence alignment of ovine *KRTAP36-2*. The four variant sequences (KRTAP36-2*A to KRTAP36-2*D) discovered in this research were aligned with the sheep genome assembly (NC_056054.1) and a representative example (GO776873.1) of the ESTs listed in Supplementary Table S1. Nucleotides in the coding region are displayed in upper case, and those outside the coding region are in lower case. Dashes indicate nucleotides matching the top sequence, and the nucleotide differences are shown with the positions indicated above the sequences. The putative TATA box, the transcription start site (TSS), and the start and stop codons are displayed in bold.

The PCR-SSCP analysis of ovine *KRTAP36-2* revealed four unique banding patterns (named variants *A* to *D*), with either one or a combination of two patterns being observed in each sheep. This indicated that the sheep were either homozygous or heterozygous (Figure 3). The sequencing of PCR amplicons representative of the distinctive banding patterns revealed four different nucleotide sequences with a length of 361 base pairs (variant *A*) or 363 base pairs (variants *B*–*D*). Of these, the sequence of *B* was identical to the sheep assembly sequence NC_056054.1, and sequences *A* and *D* were identical to the 19 ovine ESTs described above within the region transcribed (Figure 4). These variant sequences were submitted to GenBank with accession numbers OR684903-OR684906.

Overall, four single-nucleotide polymorphisms (SNPs) and a di-nucleotide deletion were detected in ovine *KRTAP36-2* (Figure 4). One of the SNPs was located downstream of the presumed TATA box, and the two-nucleotide deletion was near the transcription initiation site. The remaining three SNPs were located within the coding region, and one of them (c.79T/G) would result in an amino acid change (p.Cys27Gly) near the middle of the protein.

Eight genotypes (*AA*, *AB*, *AC*, *AD*, *BB*, *BC*, *BD*, and *CC*) were detected in the 170 sheep used for variation screening. Variant *A* was the most common and occurred at a frequency of 76.5%, and variants *B*, *C*, and *D* were only detected at frequencies of 10.3%, 8.8%, and 4.4%, respectively.

### 3.3. Association of KRTAP36-2 Variation with Wool Traits

In the 274 Southdown × Merino lambs, eight genotypes were found, namely: *AA* (n = 83), *AB* (n = 101), *AC* (n = 26), *AD* (n = 13), *BB* (n = 20), *BC* (n = 18), *BD* (n = 11), and *CD* (n = 2). This gave variant frequencies of 55.9%, 31.0%, 8.4%, and 4.7% for *A*, *B*, *C*, and *D*, respectively.

As variant *D* occurred at a frequency of less than 5.0%, those lambs carrying variant *D* were eliminated from the association analyses given the likelihood that this small group might bias any assessment. Associations were accordingly only examined in the remaining 248 lambs for variants *A*, *B*, and *C*.

Of the ten wool traits investigated, an association was only detected for the presence/absence of variant *A* and variation in YIELD. The presence of *A* was found to be associated with a decrease in YIELD (present: 74.2 ± 2.04%; absent: 78.3 ± 2.49%; *p* = 0.008). A trend of association was observed for variant *B* with MFC and variant *C* with MSS (Table 1), but no other patterns were revealed.

## 4. Discussion

This study identified a novel gene in sheep and reported variation in the gene that may be associated with some wool traits, albeit in a selected population derived from five sires and only two breeds. Several lines of evidence support the conclusion that the new gene is a *KRTAP*. First is the structure and size of the gene. The new gene appears to contain only one coding exon with a short ORF of only 174 bp. This compares well with the observation that all the *KRTAPs* identified to date are both intron-less and small (with a coding region of usually less than 800 bp) [16]. Second is the chromosomal location of the new gene. To date, the *KRTAPs* have been found to be clustered in one of five different chromosome regions [16]. In this respect, the new gene is in the region of ovine chromosome 1, where many previously identified *KRTAPs* have been found. Third is the sequence composition of the protein that the ORF would potentially produce if expressed. The KAP proteins characteristically have a high content of either cysteine or glycine and tyrosine [7]. The new gene encodes a glycine/tyrosine-rich protein, and the proportion of glycine and tyrosine residues is in the range of 35–60 mol% reported for previously identified HGT-KAPs. Lastly is the presence of ESTs that are similar or identical to the novel gene, and the tissue source from which the ESTs are derived. Because the KAPs are a structural component of wool fibres, the KAP genes are expected to be expressed in skin/wool follicles. The isolation of ESTs that have sequences identical to variants of this new gene from sheep skin and wool follicles suggests that the gene is expressed, and that when expressed, it is expressed in skin/wool follicle tissue, in accordance with the location of expression expected for KAP genes. It will however need to be confirmed that the *KRTAP36-2* sequence produces a unique amino acid sequence.

The association detected with ovine *KRTAP36-2* was different to that reported for its closely related gene *KRTAP36-1*, despite the genes being approximately 7.9 kb apart. With this same population of sheep, an association was described between variation in *KRTAP36-1* and variation in PF [8]. Despite an association with wool yield having been reported for *KRTAP15-1* [19], *KRTAP20-1* [20], *KRTAP21-1* [21], and *KRTAP22-1* [22], these genes are located further away on chromosome 1 than *KRTAP36-1* is. This suggests that the association detected for *KRTAP36-2* may reflect the effect of the gene in isolation and is unlikely to be due to tight linkage to nearby *KRTAPs*. The verification of this finding and the potential for linkage will require further investigation in a larger population that has both genetic diversity and detailed phenotypic data.

The detection of association between variant *A* and wool yield suggests that variation in ovine *KRTAP36-2* may in some way affect the quantity of non-fibre wool components and consequently affect wool yield. It is difficult to understand how such an effect may arise, but it is possible that *KRTAPs* may affect a fibre property or properties that are not routinely measured, yet indirectly affect the amount of non-fibre components in unscoured wool. There are two possible fibre properties that may lead to this effect.

The first is the surface-area-to-volume ratio of the fibres. Variation in the diameter and shape of fibres will affect this ratio, with fibres that are smaller in diameter having a higher surface-to-volume ratio. Fibres that have a more elliptical cross-section will also have an increased surface-area-to-volume ratio. An increased ratio of surface area to volume may therefore provide a greater surface area on which wool wax, suint, and vegetable matter may accumulate, thus affecting the yield [19]. While the presence of more elliptical fibres may also lead to an increase in FDSD and CVFD, this was not observed in the current study, suggesting that the ratio of surface area to volume is less likely to be the cause.

The second property that may explain the variation in yield is the ability of the fibre to absorb water. Wool is hygroscopic and can absorb up to 30% of its weight before it begins to feel wet. At 65% relative humidity, the amount of absorption is between 13% and 18% [23]. The ability of individual wool fibres to absorb water is not uniform and may be affected by several factors. Among these, one factor is the ratio of paracortex to orthocortex in individual fibres. Wool fibres have a bilateral structure with an orthocortex on one side and a paracortex on the other. The orthocortex and paracortex have different compositions, with a higher cysteine content being found in the paracortex, but a higher content of tyrosine, phenylalanine, and glycine present in the orthocortex [24]. This suggests that HS- and UHS-KAPs are preferentially present in the paracortex, and the HGT-KAPs are preferentially present in the orthocortex. The difference in protein and amino acid composition would make the paracortical and orthocortical cells swell at different rates and to different degrees when the fibre is exposed to moisture. Accordingly, any variation in the level of expression of an HGT-KAP (or HS- and UHS-KAP) could affect water absorption and thus fibre weight relative to how much wool wax, suint, and vegetable matter has accumulated.

In this respect, variant *A* differed from other variants at SNP c.-75C/A and with the di-nucleotide deletion c.-43_-42delAA. The detection of an association between yield and variant *A* suggests that these SNPs and/or the di-nucleotide deletion may have a functional effect. SNP c.-75C/A is located near to the putative TATA box, and the di-nucleotide deletion is in the 5′ untranslated region (5′ UTR). While TATA-flanking sequences do not contact the TATA-binding protein (TBP) directly, they can influence TBP affinity as well as the level of basal and activated transcription [25]. The 5′ UTR is the site that allows the ribosome scanning of the mRNA for a suitable translational start codon to initiate translation. Prior studies have suggested that variations in the 5′ UTR can influence gene expression and alter the amount of protein produced at translation [26,27], but the next step in this work might be to ascertain whether 5′ UTR variation affects RNA polymerase binding or transcription initiation.

## 5. Conclusions

This study identified a new HGT-KAP gene called *KRTAP36-2* in sheep and revealed four variant sequences resulting from four SNPs and a dinucleotide deletion in the gene. Variation in ovine *KRTAP36-2* was found to be associated with wool yield, suggesting that the gene affects the quantity of non-fibre wool components, but the mechanisms behind this require further investigation.

## Figures and Tables

**Table 1:** Association of ovine *KRTAP36-2* variants and wool traits.

Wool Trait ^1^	Variant Assessed	Absent		Present		*P* ^3^
		Mean ± SE ^2^	n	Mean ± SE	n	
GFW (kg)	*A*	2.5 ± 0.16	38	2.4 ± 0.14	210	0.124
	*B*	2.4 ± 0.15	109	2.4 ± 0.14	139	0.295
	*C*	2.4 ± 0.15	204	2.5 ± 0.17	44	0.255
CFW (kg)	*A*	1.9 ± 0.15	38	1.8 ± 0.13	210	0.861
	*B*	1.8 ± 0.12	109	1.8 ± 0.12	139	0.647
	*C*	1.8 ± 0.13	204	1.9 ± 0.15	44	0.575
YIELD (%)	*A*	77.3 ± 2.44	38	74.2 ± 2.08	210	**0.029**
	*B*	74.7 ± 2.21	109	74.1 ± 2.18	139	0.632
	*C*	74.9 ± 2.13	204	73.0 ± 2.43	44	0.233
MSL (mm)	*A*	96.8 ± 4.86	38	97.6 ± 4.16	210	0.773
	*B*	97.4 ± 4.33	109	97.6 ± 4.28	139	0.915
	*C*	97.4 ± 4.21	204	98.1 ± 4.75	44	0.805
MSS (N/ktex)	*A*	26.3 ± 3.50	38	27.0 ± 3.00	210	0.717
	*B*	27.1 ± 3.11	109	26.8 ± 3.08	139	0.848
	*C*	25.9 ± 2.99	204	29.2 ± 3.37	44	*0.070*
MFD (µm)	*A*	18.5 ± 0.55	38	18.3 ± 0.48	210	0.494
	*B*	18.2 ± 0.49	109	18.4 ± 0.48	139	0.322
	*C*	18.1 ± 0.52	204	18.6 ± 0.52	44	0.237
FDSD (µm)	*A*	3.5 ± 0.22	38	3.5 ± 0.19	210	0.610
	*B*	3.5 ± 0.18	109	3.5 ± 0.18	139	0.628
	*C*	3.5 ± 0.18	204	3.6 ± 0.20	44	0.386
CVFD (%)	*A*	19.1 ± 0.97	38	19.0 ± 0.83	210	0.949
	*B*	19.4 ± 0.87	109	18.9 ± 0.86	139	0.182
	*C*	19.1 ± 0.86	204	19.1 ± 0.95	44	0.997
PF (%)	*A*	0.5 ± 0.51	38	0.7 ± 0.43	210	0.442
	*B*	0.8 ± 0.45	109	0.6 ± 0.44	139	0.596
	*C*	0.6 ± 0.44	204	0.9 ± 0.50	44	0.428
MFC (°/mm)	*A*	72.1 ± 6.80	38	78.0 ± 5.78	210	0.128
	*B*	80.8 ± 5.99	109	74.9 ± 5.92	139	*0.063*
	*C*	76.9 ± 5.89	204	80.1 ± 6.66	44	0.447

^1^ GFW—greasy fleece weight; CFW—clean fleece weight; YIELD—wool yield; MSL—mean staple length; MSS—mean staple strength; MFD—mean fibre diameter; FDSD—fibre diameter standard deviation; CVFD—coefficient of variation of fibre diameter; PF—prickle factor (percentage of fibres over 30 μm); MFC—mean fibre curvature. ^2^ Predicted means and standard errors of those means derived from the GLMs. ^3^ *p* values < 0.05 are in bold, and *p* values < 0.10 are italicised.

## Data Availability

The original data used in this paper are available by contacting the corresponding author upon request.

## Supplementary Materials

The following supporting information can be downloaded at: https://www.mdpi.com/article/10.3390/genes14112045/s1, Table S1: Ovine EST sequences showing 99% sequence identity to the ORF identified in this study.
