# Supplementary material for: Ovine KRTAP36-2: A New Keratin-Associated Protein Gene Related to Variation in Wool Yield
Source: Genes (Basel). 2023 Nov 6;14(11):2045. doi: 10.3390/genes14112045 (PMC10671549; doi:10.3390/genes14112045)
Supplement: Supplementary file 1 [file genes-14-02045-s001.zip › genes-2635406-supplementary.pdf]

Supplementary Table S1. Ovine EST sequences showing 99% sequence identity to the ORF identified in this study.

**Description**	**Scientific Name**	**Max Score**	**Total Score**	**Query Cover**	**E value**	**Percent identity**	**Acc. Len**	**GenBank Accession Number**
010801OFSA032713HT OFSA Ovis aries cDNA 5', mRNA sequence	*Ovis aries*	311	311	100%	1.00E-81	99	516	GO776873.1
010709OFSA028031HT OFSA Ovis aries cDNA 5', mRNA sequence	*Ovis aries*	311	311	100%	1.00E-81	99	519	GO745965.1
010709OFSA027638HT OFSA Ovis aries cDNA 5', mRNA sequence	*Ovis aries*	311	311	100%	1.00E-81	99	518	GO748537.1
000905OASA004179HT OASA Ovis aries cDNA 5', mRNA sequence	*Ovis aries*	311	311	100%	1.00E-81	99	530	GO734707.1
001114OFSA012892HT OFSA Ovis aries cDNA 5', mRNA sequence	*Ovis aries*	311	311	100%	1.00E-81	99	343	GO710657.1
010704OFSA026838HT OFSA Ovis aries cDNA 5', mRNA sequence	*Ovis aries*	311	311	100%	1.00E-81	99	514	GO711704.1
010702OFSA025652HT OFSA Ovis aries cDNA 5', mRNA sequence	*Ovis aries*	311	311	100%	1.00E-81	99	214	GO679356.1
001114OFSA012910HT OFSA Ovis aries cDNA 5', mRNA sequence	*Ovis aries*	311	311	100%	1.00E-81	99	330	GO674524.1
030729OSPA2029078HT OSPA Ovis aries cDNA, mRNA sequence	*Ovis aries*	311	311	100%	1.00E-81	99	498	EE854050.1
030729OSCA3004013HT OSCA Ovis aries cDNA, mRNA sequence	*Ovis aries*	311	311	100%	1.00E-81	99	494	EE849207.1
030522OSCA3002013HT OSCA Ovis aries cDNA, mRNA sequence	*Ovis aries*	311	311	100%	1.00E-81	99	453	EE848242.1
030729OSCA3023065HT OSCA Ovis aries cDNA, mRNA sequence	*Ovis aries*	311	311	100%	1.00E-81	99	492	EE848165.1
030729OSCA3010040HT OSCA Ovis aries cDNA, mRNA sequence	*Ovis aries*	311	311	100%	1.00E-81	99	521	EE848138.1
010914OSAA043090HT OSAA Ovis aries cDNA, mRNA sequence	*Ovis aries*	311	311	100%	1.00E-81	99	284	EE839651.1
020605OCS411011005HT OCS4 Ovis aries cDNA, mRNA sequence	*Ovis aries*	311	311	100%	1.00E-81	99	463	EE755445.1
020502OCSI1017061HT OCS1 Ovis aries cDNA, mRNA sequence	*Ovis aries*	311	311	100%	1.00E-81	99	483	EE753108.1
020314OCSI1001052HT OCS1 Ovis aries cDNA, mRNA sequence	*Ovis aries*	311	311	100%	1.00E-81	99	495	EE751850.1
020502OCSI1020011HT OCS1 Ovis aries cDNA, mRNA sequence	*Ovis aries*	311	311	100%	1.00E-81	99	492	EE751738.1
020502OCSI1019082HT OCS1 Ovis aries cDNA, mRNA sequence	*Ovis aries*	311	311	100%	1.00E-81	99	495	EE751673.1
